# Risk Factors of Lymph Edema in Breast Cancer Patients

**DOI:** 10.1155/2013/641818

**Published:** 2013-06-05

**Authors:** Haghighat Shahpar, Akbari Atieh, Ansari Maryam, Homaei Shandiz Fatemeh, Najafi Massoome, Ebrahimi Mandana, Yunesian Masud, Mirzaei Hamid Reza, Akbari Mohammad Esmaeil

**Affiliations:** ^1^Breast Cancer Research Group, Breast Cancer Research Center (BCRC), ACECR, Tehran 1315685981, Iran; ^2^Cancer Research Center (CRC), Shahid Beheshti University of Medical Sciences, Tehran 1989934148, Iran; ^3^Solid Tumor Treatment Research Center, Mashhad University of Medical University, Mashhad 91397, Iran; ^4^Cancer Institute, Tehran University of Medical Sciences, Tehran 1419733141, Iran; ^5^Tehran University of Medical Sciences, Tehran 1411717415, Iran; ^6^Cancer Research Center, Shohada Hospital, Tajrish Square, Tehran, Iran

## Abstract

*Background*. Lymphedema secondary to breast cancer treatment is a common and serious problem for disease survivors. The objective of the current study was to identify the risk factors of secondary lymphedema after breast carcinoma treatment. *Materials & Methods*. The breast cancer patients who were followed up in three centers in Tehran and Mashhad in 2010 were recruited in the study. The circumference measurement was used for defining lymphedema. *Results*. Among 410 breast cancer patients, 123 cases (30%) developed lymphedema. Variables such as low educational level, body mass index (BMI), higher stage of disease, number of involved lymph nodes, comorbid diseases, trauma, infection, and the time after surgery showed significant correlation with the development of lymphedema. In logistic regression analysis, increase of 1 kg/m^2^ in BMI (OR = 1.09; 95%  CI 1.05–1.15), each number increase in lymph node involvement (OR = 1.15; 95%  CI 1.08–1.21) and the increase of every 1 month after surgery (OR = 1.01; 95%  CI 1.01–1.02) significantly increased the risk of lymphedema. *Conclusion*. The results of this study demonstrated that preserving a fitted BMI, emphasis on self-care, and educating preventive activities may have important roles in decreasing the lymphedema incidence and improving the patients' quality of life.

## 1. Introduction

Lymphedema (LE) is an external (or internal) manifestation of lymphatic system insufficiency and impaired lymph transport [1]. It is characterized by accumulation of lymphatic fluid in the interstitial tissue that causes swelling, most often in extremities.

The development of LE occurs when the lymphatic load exceeds the transport capacity. There are two general classifications of LE: primary and secondary. Primary LE develops as a consequence of a pathologic congenital and/or hereditary etiology. Secondary LE, which is more common, is caused by mechanical insufficiency of the lymphatic system due to surgery, radiation, chemotherapy, trauma, infection, tumoral blockage, chronic venous insufficiency, immobility, or tourniquet effects [2].

Upper extremity LE is one of the most common complications after breast cancer surgery with a reported incidence of 6% to 30% [3]. It is estimated that 120,000–600,000 patients suffer from postmastectomy lymphedema in the United States [4]. In a meta-analysis of 72 studies achieved by Disipio et al. in 2013, pooled estimate of lymphedema incidence was 16.6% (95% CI 13.6–20.2). It was 21.4% (14.9–29.8) when data were restricted to prospective cohort studies (30 studies) [5]. 

The occurrence time of lymphedema has been studied in many researches. The incidence of arm lymphedema seems to increase up to 2 years after diagnosis or surgery of breast cancer [5]. Its overall incidence ranges from 8% to 56% at 2 years of followup, depending on the extent of axillary surgery and the use of radiotherapy [6].

In recent decades, breast cancer mortality rates have declined, reflecting advances in early detection as well as more widespread application of effective adjuvant therapies, and many women diagnosed with breast cancer may expect survival probability similar to age-matched women without breast cancer. The National Cancer Institute estimates that 1 in 7 women in the USA have a lifetime risk of being diagnosed with BC and 1 in 33 will die from it [7]. As life expectancy improves for women with breast cancer, more women will be living with possible side effects of the treatment. Consequently, effective prevention and management of treatment sequels such as lymphedema that can impair function and quality of life in breast cancer survivors have taken on increasing importance [8]. 

Lymphedema may present with different signs and symptoms including a feeling of heaviness or tightness in the limb, pain or discomfort, restricted range of motion, and swelling in a part or entire limb. 

 Several variables have been identified as potential risk factors for development of breast cancer related lymphedema. In a study on breast cancer patients living in Florence, radiotherapy, the number of lymph nodes removed, and the size of the tumor were identified as significant prognostic factors that increase the risk of lymphedema in patients who undergo dissection of the axillary lymph nodes [9].

 According to a review conducted by American Cancer Society, the most important risk factors for the development of lymphedema are tumor location in the upper outer quadrant, postoperative axillary trauma, infection, hematoma, and seroma, axillary radiation after axillary lymph node dissection (ALND), extent of ALND (inclusion of level 3), axillary recurrence, and large number of positive axillary lymph nodes [3]. Meta-analysis of 29 studies showed that risk factors with strong level of evidence were extensive surgery (i.e., axillary lymph node dissection, greater number of lymph nodes dissected, and mastectomy) and being overweight or obese [5].

The aim of this study was to examine the main lymphedema risk factors in breast cancer patients referred to 3 cancer centers in Iran. Defining the most important risk factors of lymphedema can help physicians and health care providers to explain precautions to the patients and encourage them to seek care promptly if they experience symptoms of lymphedema and promote the surgeons to choose more safe and conservative treatments.

Importantly, with the early identification and management of lymphedema, we can help patients to maintain their quality of life by minimizing cosmetic, functional, psychoemotional, and potentially life-threatening complications.

## 2. Materials and Methods

### 2.1. Study Population

Sample size of this study was estimated by Epi info software to be about 400 patients. This calculation was based on 80% power and 5% of two sided significance level (alpha error) and reported OR of most important lymphedema risk factors in previous studies.

The breast cancer patients who were being followed up less than 3 years after initial treatment in Breast Cancer Research Center (Tehran), Azar Sample from Cancer Research Center (Tehran) and Ghaem Hospital (Mashhad) in 2010 were included in the study. Patients who had bilateral breast cancer and male cases were excluded from the study. This project was approved by ethical committee of BCRC. 

### 2.2. Data Collection

Demographic and clinical characteristics of patients referred to 3 centers for followup were recorded by a questionnaire. Data consisted of the following.

1. Demographic variables (age, marital status, educational status, and dominant hand) and past medical history (history of comorbid conditions such as diabetes, hypertension, chronic heart failure, renal failure, hypothyroidism, and history of infection or trauma in affected limb) were collected by interview with participants.
2. Treatment modalities consisting of the type of surgery, chemotherapy regimen, radiotherapy, and hormone therapy were identified by patient interview and physicians' notes. Data pertaining to the tumor size, number of excised and involved lymph nodes were recorded according to pathology report.
3. BMI: height and weight were measured by a trained observer in interview, and BMI was defined as the weight in kilograms divided by the square of the height in meters.

Arm circumferences were measured at 5 points in two arms. These points were hand (at the first and fifth metacarpal), wrist (the distal edge of the styloid process), 10 cm below elbow, and 5 and 15 cm above elbow. If the circumference difference was 2 cm or higher in any point, she was considered as a lymphedema case. Their characteristics were compared to other patients without edema, with the difference of less than 2 cm in any point. 

Some subjective symptoms were also recorded. Women were asked to answer “yes” or “no” to the questions about having arm symptoms (pain, heaviness, and paresthesia) and their experience of edema. Their answer to the question “since the diagnosis of breast cancer have you experienced arm swelling?” indicated the presence of subjective edema. 

### 2.3. Data Analysis

By univariate analysis, the independent effect of each variable on edema occurrence was studied. Logistic regression was used to examine the intercorrelation effect of demographic and clinical characteristics on outcome of interest. Two-tailed test and *P* value <0.05 were considered as statistical significance values. Considering the important role of age, physical activity, and number of excised lymph nodes, as were mentioned in many previous studies, variables with significance level of *P* ≥ 0.1 were included in regression analysis. 

The frequencies of subjective symptoms in two groups were compared by kappa coefficient. In general, values of kappa greater than 0.80 denote very good agreement beyond chance, values below 0.2 indicate poor agreement, and values between 0.2 and 0.4, 0.4 and 0.6, and 0.6 and 0.8 represent fair, moderate, and good agreement beyond chance, respectively [10].

Statistical procedures were performed using the statistical package SPSS 17 for windows. 

## 3. Results

Totally, 410 breast cancer patients were studied. According to lymphedema definition, 123 patients (30%) had lymphedema and were defined as cases. All the other 298 patients were considered as noncase group. The mean age of the patients was 49 years (±10.9). Half of them were educated high school and higher, and about 88% of them were married.

Demographic and clinical characteristics of patients (Table 1) showed that the educational level of cases was significantly lower than the other group (*P* = 0.02). In 58.5% of patients with lymphedema, the dominant arm and affected one were the same while in noncase group this proportion was 62%. Breast cancer in advanced stages was more common in cases (24.4%) compared to the second group (14.6%), and the frequency of trauma and comorbid diseases in lymphedematous patients compared to noncases were 9.8% versus 3.8% and 35% versus 24%, respectively.

Table 2 shows the mean difference of age, BMI, number of excised and involved lymph nodes, and the follow-up duration between two groups. The effect of each unit change of these variables on increasing the odds of lymphedema occurrence has been measured by odds ratio. It is noticeable that half of the lymphedema had been occurred in the first 17 months after surgery.

Categorical and continuous variables on lymphedema incidence were analyzed by logistic regression. Predictor factors of this analysis and their effect have been displayed in Table 3. 

As a secondary outcome, comparison of subjective edema and symptoms between two groups was achieved (Table 4). The agreement level of subjective symptoms with objective ones was assessed by kappa coefficient.

 In 410 patients surveyed, 130 (31.7%) reported having developed arm swelling after their treatment and about 39% of them complained of symptoms such as pain, heaviness, and paresthesia. The frequency of these symptoms in cases compared to noncases was 47.2 versus 35.5 percent (*P* = 0.018). 

## 4. Discussion

Lymphedema is a common complication of cancer therapy. It can occur anywhere that lymph nodes have been surgically removed or lymph flow has been disturbed.

The results of this study showed that each unit increase of BMI, every additional lymph node involvement, and each month after surgery could increase the odds of swelling by 9%, 15%, and 1%, respectively.

Swelling may occur at any point following axillary node dissection or radiation therapy, beginning immediately after or even delayed by several years. Our findings revealed the incidence of 30% for lymphedema among breast cancer survivors who were studied with a mean followup of 34 months in lymphedema group. 

Lymphedema following treatment for breast cancer has received attention in multiple studies. According to some reports, overall incidence of arm lymphedema can range from 8% to 56% in 2 years following surgery, depending on the extent of axillary surgery and the use of radiotherapy. It develops most commonly in the first 12 to 14 months following treatment [6]. In this study, half of the lymphedema had been detected in the first 17 months after surgery which was similar to other studies. So, more frequent surveillance throughout this time (e.g., once every 3–6 months) seems reasonable [5]. In Petrek et al. study, the onset of lymphedema was noted by approximately three-quarters (77%) of the patients within the first 3 years after breast cancer treatment [11]. The large range in the prevalence can be due to various methods of edema measurement [12, 13], and this highlights the importance of determining an accurate measurement method by future studies.

Similar to some other studies [14], we did not find any significant statistical correlation between marital status and lymphedema, while Paskett and colleagues have shown that the risk of swelling among married women was 1.36 times higher than the risk for unmarried women [6].

Univariate analysis of demographic characteristics showed that lymphedema was significantly more prevalent in higher age, lower levels of education, and higher BMI. It was shown that the odds of edema in illiterates and low levels of education was 1.66 times more than higher levels of education. Definitely higher awareness and better self-care practice in educated patients have an important role in this difference. 

In spite of some studies that neither age at the time of assessment nor age at the diagnosis of breast cancer was predictor of lymphedema [15], Meeske et al. have shown that arm lymphedema was associated with younger age at diagnosis (OR per year of age = 0.96, CI = 0.93–0.99), history of hypertension (OR = 2.31, CI = 1.38–3.88), obesity (OR = 2.48, CI = 1.05–5.84) and 10 or more lymph node excised (OR = 2.16, CI = 1.12–4.17) [16]. In a study in Hong Kong, adjusted odds ratios for the development of lymphedema were 1.11 (95% CI = 1.01–1.22) for an increase of 1 kg/m^2^ in body mass index (BMI) at recruitment and 1.05 (95% CI = 1.01–1.10) for an increase of 1 year of age at recruitment time [17]. Hayes showed that age more than 50 may increase the risk of lymphedema incidence to 3.3 times [18].

It seems that in older people, lower physical activity, weakness of muscle pump, and higher BMI may increase edema. 

The association between BMI and lymphedema has been approved in many studies [3, 13, 15]. Soran et al., believe that it is not clear whether obesity is a direct risk factor for arm edema; it is certainly a risk factor for infection and poor wound healing [4]. Foeldi believes that lymphedema combined with obesity is more than the sum of the two diseases. Obesity causes the diaphragm to be above its normal position, impairing its movement. As a result, a mechanism that supports lymph flow is impaired [19]. Segerstrom et al. (1992) surmised that increased weight may lead to an increase in radiation dose, which may be associated with lymphedema. So they suggest that controlling BMI can be helpful in reducing lymphedema, even though it may not be a direct risk factor for it [8]. According to mentioned evidences it seems that all physicians and health care providers should strongly encourage breast cancer patients to engage routinely in weight control strategies to minimize their risk for swelling and development of other chronic diseases.

Treatment modalities have been introduced as predictor factors of lymphedema in different studies. Even though type of surgery [8, 15, 20] chemotherapy [6, 13] and radiotherapy [2, 8, 9, 13] has shown significant relation with the incidence of edema, but such an association have not been insisted in some studies [8, 11]. According to recent meta-analysis, strong evidence supports the association of extensive surgery (chest wall and axilla) with increased risk of lymphedema and moderate evidence supports its association with adjuvant therapy (radiation and chemotherapy) [5]. In this research, none of those treatment modalities showed significant relation with lymphedema but the limitations of data collection in this study should not be overlooked. For example, data related to direct radiation to the axilla had not been recorded, and the possibility of radiation scattered to the axilla during breast or chest wall therapy could not be estimated. 

Patients underwent surgery by several surgeons with different techniques of surgery, and this could confound the comparison of role of modified radical mastectomy with breast preservation in inducing edema. According to a research referred by Foeldi, lymphedema develops more frequently after breast cancer surgeries performed by residents compared to surgeries performed by experienced surgeons [19]. Thus, the experience of the surgeon and less manipulation are important factors for decreasing the risk of lymphedema.

Risk reduction related to surgeons' experience can be reflected specifically in axillary dissection. It is expected that the use of sentinel lymph node biopsy (SLNB) as a minimally invasive technique instead of classical axillary lymph node dissection can reduce the incidence of lymphedema.

In spite of many studies which have introduced the higher number of excised lymph nodes as a predictor of lymphedema [3, 6, 9, 16, 21], in some valid studies only a higher number of involved lymph nodes or a higher stage of disease were noticed to be related to lymphedema [2, 13]. In our study, the number of the involved lymph nodes was represented as edema risk factor (OR = 1.15, CI = 1.08–1.21), revealing that for every node involved, the odds of swelling increased by 15%. It is expected that by early diagnosis of breast cancer in lower stage, the incidence of lymphedema would decline.

As we mentioned before, a subjective comparison of feeling edema and lymphedema symptoms between two groups was made. About 31.7% of patients reported developing arm swelling after their treatment, and 39% of them complained of symptoms such as pain, heaviness, and paresthesia. In a study of Otago women, the frequency of subjective edema and pain has been reported 38% and 37.5% in patients with and without lymphedema [22]. Pain is a common symptom in lymphedema patients. For example, in Paskett and Stark study 72% of the lymphedema patients reported pain in addition to edema, and 57% of them had intermittent pain [23], and Moffatt showed that 50% of patients had experienced pain or discomfort from their edema [24]. In our study, 47.2% of lymphedema patients complained of arm symptoms.

In the present study, the agreement between subjective edema with measured lymphedema and arm symptoms was considered as indexes of patients' knowledge of lymphedema. The result of analysis showed fair and poor level of agreement coefficient between the current variables (0.33 versus 0.15). It is noticeable that about 60% of patients with arm symptoms and 44.7% of cases had no complaint of edema. The results of a study found that the symptoms of “heaviness in the past year” and “swelling now” were predictive of a maximal limb circumference difference of 2 cm, so they introduce these symptoms as precursors to the clinical diagnosis of lymphedema [2]. The low level of agreement in our study can emphasize that many patients are not familiar with the early signs and symptoms of lymphedema. So, educational programs may be helpful and necessary for increasing knowledge of patients and healthcare practitioners for providing better self-care and preventive strategies. 

One of the advantages of this study is recruitment of study sample in follow-up clinics. Mostly the main protocol of these clinics does not focus on lymphedema, besides recording patients' data before measurement of arm circumference could decrease recall bias. These three clinics are referral centers for breast cancer in two cities of Iran. So, considering that they are not representative of all breast cancer patients in Iran, but this design provided an opportunity for estimating the frequency of lymphedema in referred patients.

In this study, about 62% of patients whose dominant hand and involved limb were the same had no defined lymphedema. Even though in some studies the disease in dominant side has been introduced as a predictor factor for lymphedema [17], there are other researches that have not shown such an association [8]. The authors of this paper believe that after surgery, most of the patients automatically limit the movement of the affected side which can induce edema. If this side is the dominant hand, the patient automatically uses it in daily activities, and by increasing the lymph flow, the incidence of lymphedema may decrease.

As many other studies, we had the limitation in measurement method validity which was unavoidable in spite of the training of the observers. 

Another limitation was natural asymmetry of healthy limbs. As we know, there can be a slight natural difference between a subject's arms because of hypertrophied muscle in the overused arm. For studying the effect of this variable, the comparison between the preoperative and postoperative measurement of the same arm could be a more precise method. So it is recommended that lymphedema assessment should begin preoperatively with assessment of both arm, and it ought to be continued at regular intervals.

## 5. Conclusion

According to the finding of this study, it seems that preserving a fitted BMI, increasing patients' knowledge about lymphedema, emphasis on self-care, and educating preventive strategies after surgery and during follow-up visits may have important roles in decreasing the lymphedema incidence. 

To achieve this goal and improving quality of life of patients, educating health professionals regarding basic pathophysiology of lymphedema and promoting early diagnosis and treatment modalities may be beneficial practices.

## Figures and Tables

**Table 1:** Demographic and clinical characteristic of patients.

Variable	Case no. (%)	Noncase no. (%)	OR (95% CI)	*P* value
Education				
Illiterate/primary school	72 (58.5)	132 (46)	1.66 (1.08–2.54)	0.02
High school/university	51 (41.5)	155 (54)	1	
Marital status				
Single/widow/divorce	14 (11.4)	34 (11.8)	0.96 (0.49–1.85)	0.893
Married	109 (88.6)	253 (88.2)	1	
Dominant limb = involve limb				
Yes	72 (58.5)	178 (62)	0.87 (0.56–1.33)	0.508
No	51 (41.5)	109 (38)	1	
Physical activity				
Low	34 (27.6)	54 (18.8)	1.89 (0.95–3.75)	0.069
Moderate	71 (57.7)	179 (62.4)	1.19 (0.65–2.17)	0.57
High	18 (14.6)	54 (18.8)	1	
Stage of disease				
I	10 (8.1)	37 (12.9)	1	
II	83 (67.5)	208 (72.5)	1.48 (0.7–3.1)	0.304
III/IV	30 (24.4)	42 (14.6)	2.64 (1.14–6.13)	0.024
Breast surgery				
Modified radical mastectomy	83 (67.5)	197 (68.6)	0.95 (0.6–1.49)	0.817
Breast preservation	40 (32.5)	90 (31.4)	1	
Radiation therapy				
No	31 (25.2)	88 (30.7)	1	
Yes	92 (74.8)	199 (69.3)	1.32 (0.81–2.12)	0.265
Chemotherapy				
No	7 (5.7)	13 (4.5)	1	
Regimen with Adriamycin	95 (77.2)	214 (74.6)	0.82 (0.31–2.13)	0.69
Regimen without Adriamycin	21 (17.1)	60 (20.9)	0.65 (0.23–1.85)	0.42
Hormone therapy				
No	50 (40.7)	119 (41.5)	1	
Yes	73 (59.3)	168 (58.5)	1.03 (0.67–1.59)	0.878
Co-morbid disease				
No	80 (65)	216 (75.3)	1	
Yes	43 (35)	71 (24.7)	1.64 (1.04–2.58)	0.035
History of Trauma/infection in affected limb				
No	111 (90.2)	276 (96.2)	1	
Yes	12 (9.8)	11 (3.8)	2.71 (1.16–6.33)	0.02
History of seroma				
No	113 (91.9)	260 (90.6)	1	
Yes	10 (8.1)	27 (9.4)	0.85 (0.4–1.81)	0.679

**Table 2:** Demographic and clinical characteristics of patients (numeric variables).

Variable	Case mean (±SD)	Non-case mean (±SD)	OR (95% CI)	*P* value
Age (years)	50.6 (±11.4)	48.4 (±10.6)	1.02 (0.99–1.04)	0.059
BMI	30.7 (±5.2)	28.8 (±4.6)	1.08 (1.03–1.13)	<0.001
Tumor size (cm)	3.1 (±1.5)	2.9 (±1.5)	1.21 (0.98–1.28)	0.98
No. of excised LN	11.1 (±5.3)	10.1 (±4.9)	1.04 (1–1.28)	0.062
No. of involved LN	4.2 (±5.2)	2 (±3.5)	1.132 (1.07–1.97)	<0.001
Duration after surgery (months)	34.2 (±38.8)	27.6 (±25.8)	1.007 (1–1.01)	0.04

**Table 3:** Predictor factors of lymphedema.

Variable	β	SE	*P*-value	OR (65% CI)
Constant	−4.17	0.75	<0.001	
BMI	0.09	0.24	<0.001	1.09 (1.05–1.15)
No. of involved LN	0.14	0.03	<0.001	1.15 (1.08–1.21)
Months after surgery	0.008	0.004	0.025	1.01 (1.01–1.02)

**Table 4:** Subjective comparison of feeling edema and lymphedema symptoms.

Variable	Frequency of subjective edema (%)		Kappa co-efficient	*P* value
	No	Yes		
Presence of lymphedema			0.33	<0.001
Noncase	225 (78.4)	62 (21.6)		
Case	55 (44.7)	68 (55.3)		
Symptoms (pain, heaviness, paresthesia)			0.15	0.002
No	185 (74)	65 (26)		
Yes	95 (59.4)	65 (40.6)		
